# How to initiate tissue regeneration by generating mutually exclusive cell states

**DOI:** 10.1371/journal.pbio.3002155

**Published:** 2023-06-06

**Authors:** Tin Tin Su

**Affiliations:** 1 Department of Molecular, Cellular and Developmental Biology, University of Colorado, Boulder, Colorado, United States of America; 2 University of Colorado Cancer Center, Anschutz Medical Campus, Aurora, Colorado, United States of America

## Abstract

A healing wound is made of cells that paused proliferation and secrete mitogenic factors and cells that proliferate to replace those lost to cell death. A new study in *PLOS Biology* describes how 2 conserved signaling pathways could repress each other to create these mutually exclusive cell states.

A healing wound is a microcosm of many cell states found during organogenesis [1,2]. In addition to dead and dying cells, one may find proliferating cells, migrating cells, and cells that are differentiating or switching fate. How is this multitude of cell states, often mutually exclusive, established and maintained for successful repair and regeneration? Jaiswal and colleagues asked this question in the context of wound healing in the *Drosophila* larval epithelia and in the context of tumors [3].

Imaginal discs of *Drosophila* larvae are made of diploid epithelial cells that differentiate into adult structures during metamorphosis. A larval wing imaginal disc is composed of a single continuous cell layer folded onto itself like pita bread. On one surface are columnar epithelial cells that will produce the adult wing, the body wall, and the hinge that connects the two. In larvae, these regions are already specified to express different genes and are called the pouch, the notum, and the hinge, respectively. Using a conditional pouch-specific gene expression system, Jaiswal and colleagues expressed Eiger (TNFα) to induce apoptosis transiently and watched the wing disc regenerate. Genetic ablation of the pouch is a powerful experimental model used by many labs. In a typical experiment, expression of death-inducers like Eiger are turned on for 24 h and then turned off to allow the start of regeneration (R0 time point) and subsequent events monitored specific hours later (R24, R48, etc.). Genetic ablation of the pouch can be compatible with full regeneration; adult flies with normal looking wings can be recovered. These studies have been extremely informative, demonstrating, for example, the role of Myc and Wg (Wnt1) in regenerative growth and transcription factor CtBP (ortholog of human CTBP1/2) in ensuring correct cell fate specification during regeneration [4,5] and documenting that hinge cells change fate to help regenerate the pouch [6].

Expression of Eiger activates JNK/AP1 signaling. Classen lab showed previously that while some pouch cells die, others arrest in the G2 phase of the cell cycle, which protects them from JNK-mediated apoptosis [7]. Though themselves arrested in G2, JNK-active cells express secreted cytokines such as Upd1-3 and can promote cell proliferation in a nonautonomous manner. Thus, the previous study detected 2 mutually exclusive cell states in the damaged pouch: JNK-active and G2 arrested versus JNK-inactive and proliferating. This is reminiscent of wound healing in mice where domains of different cellular behaviors coexist and are comprised of proliferative, migratory, or differentiating cells with different gene expression signatures [1,2]. How distinct cell states are established during epithelial wound healing in *Drosophila* is the focus of the new study [3]. To this end, the authors monitored events during Eiger expression, specifically, at 7, 14, and 24 h from the start of Eiger induction (Fig 1), the last time point being equivalent to R0.

**Fig 1:**
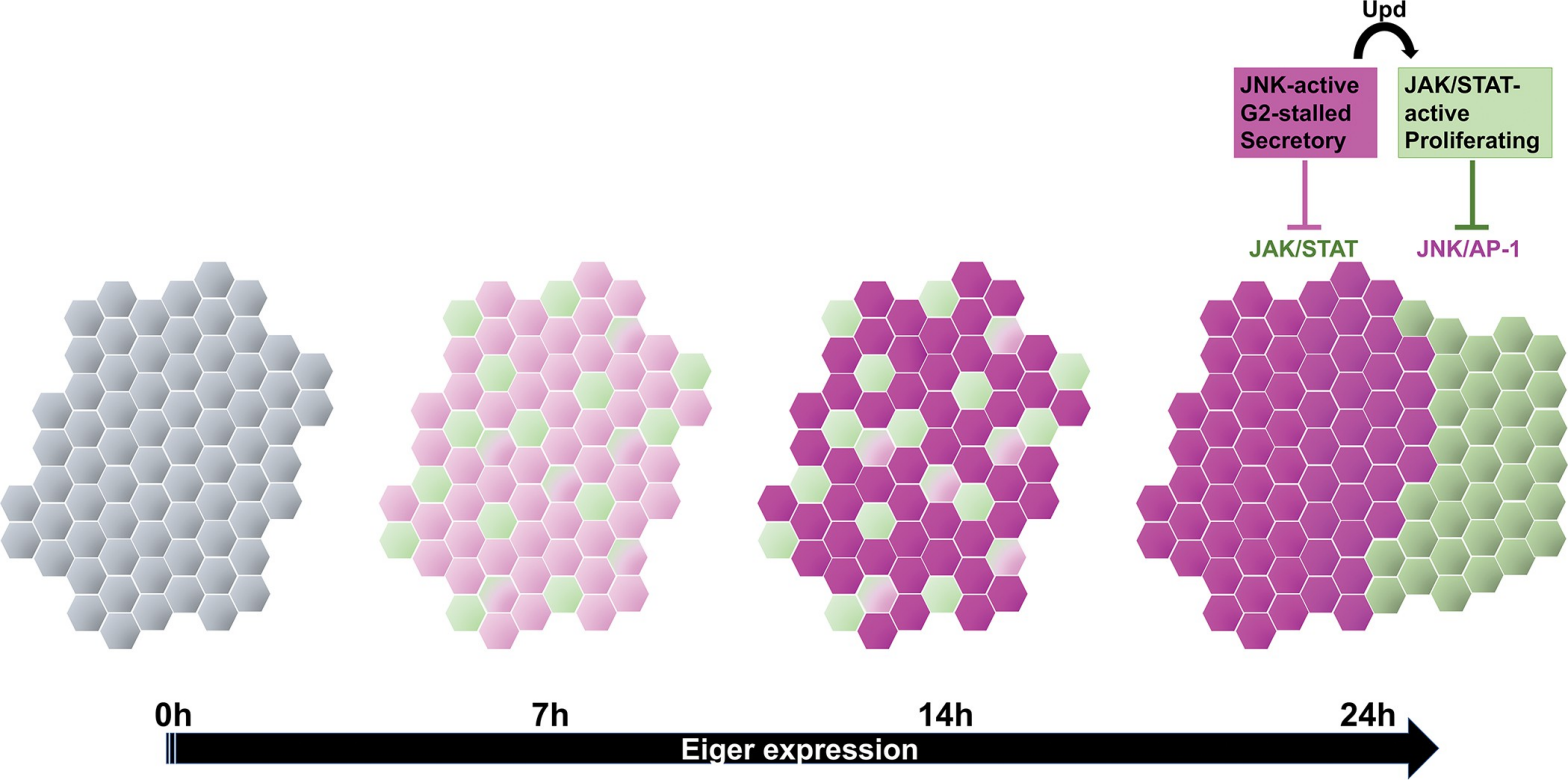
Dynamic changes in cellular states within an epithelial wound during 24 h of Eiger expression. Expression of proapoptotic TNF ortholog Eiger results initially in low level activation of JNK and JAK/STAT, with each cell activating one or both pathways. As JNK activity increases, JAK/STAT is inhibited cell-autonomously but becomes activated in the surrounding cells due to secreted Upds, cytokines that activate JAK. JAK/STAT inhibits JNK cell autonomously to create a bistable state with different cell properties.

Apoptosis, detected with an antibody to cleaved effector caspase Dcp1 (ortholog of caspase3/7), was elevated in the pouch by 7 h and persisted through later time points. Also seen in the pouch at 7 h were activation of a JNK transcriptional response reporter, an increase in cells in G2, and a decrease in cells in S phase. At 14 h, JNK activity increased further, cell cycle stalling became more prevalent, and cells started to show increasing levels of senescence-associated β-galactosidase. Ultimately, cells would also display elevated expression of Upd1-3. Interestingly, at 7 and 14 h time points, the pouch also included cells with STAT activity (ortholog of human STAT3/5) and cells with both JNK and STAT activities. Over the next 10 h, while JNK activity remained high, STAT activity declined in JNK-active cells and became elevated instead in their neighbors. Thus, 2 mutually exclusive cell states resolve between 14 and 24 h from the start of Eiger expression: JNK-active, JAK/STAT-inactive, and G2-stalled versus JAK/STAT-active and proliferating. Failure to repress JAK/STAT in JNK-active cells have dire consequences: loss of the G2 state and death by apoptosis.

The job of establishing 2 cell states appears to fall on JNK; ectopic activation of JNK by expressing an active Hep (JNK Kinase) in isolated clones of cells within an undamaged wing disc is sufficient to reproduce 2 cell states: cells with high JNK and low JAK/STAT activity that are surrounded by cells with high JAK/STAT activity. Remarkably, even in epithelial tumors generated by a combination of mutant tumor suppressor Scrib (ortholog of human SCRIB) and oncogenic Ras^V12^, cells partitioned into JNK-active/G2 and STAT-active/proliferative states despite being genetically identical. These and other data led to the model that JNK represses JAK/STAT cell-autonomously but induces JAK/STAT nonautonomously via secreted factors (Fig 1). The authors further postulated that JAK/STAT then represses JNK cell autonomously, thereby stabilizing the cell states. A mutual repression model between JNK and JAK/STAT predicted the observed bistable state robustly in mathematical modeling and is proposed by the authors to be best at preventing these signaling events from expanding beyond the wound site in an unrestrained manner.

Spatial separation of JNK and JAK/STAT activity during wound healing has been observed before, with JNK repressing JAK/STAT at the center of a wound in the larval epidermis to allow cell fusion events that are sensitive to inhibition by STAT [8]. The new study proposes an even more prominent role to the JNK-active wound center, that it acts as an organizer for regeneration, much like developmental organizers that patterns the embryo or the developing limb.

Molecular and cellular events described in the new study provide an important “prequel” to a published report of cellular organization, gene expression, and lineage relationships at R0, R24, and R48 time points in the same experimental model [9]. According to single-cell RNAseq data, regenerating cells fell into 2 groups called blastema 1 and blastema 2 that expressed several secreted proteins including cytokines Upd1-3 to form a “regenerative secretory zone.” Blastemas were surrounded by proliferating cells, much like the JNK-active core in the new study. Unlike in the new study where JNK-active cells derived from Eiger-expressing pouch cells, gene expression signatures of both blastemas suggest that they were derived from reprogrammed hinge cells. Future efforts to bridge the studies, for example, by addressing the relationship between 2 cell states in the new study and blastemas in the published study would help complete our understanding of epithelial regeneration after wounding. What we learn may be leveraged toward therapies to promote regeneration during wound healing or inhibit regeneration in the case of tumors.
